# Supplementary material for: Parental effects provide an opportunity for coral resilience following major bleaching events
Source: PLoS One. 2025 Jan 7;20(1):e0290479. doi: 10.1371/journal.pone.0290479 (PMC11706449; doi:10.1371/journal.pone.0290479)
Supplement: Attachment — Submitted filename: Review_PONE-D-23-24872.docx [file pone.0290479.s001.docx]

Review of PONE-D-23-24872

02Oct2023

*Reviewer comments are italicized and highlighted blue.

*Changes in spelling or grammar are indicated in green highlighting.

Intro:

“Coral bleaching is known to impact sexual reproduction (Baird & Marshall, 2002; Fisch et al.,

2019) and recruitment (Hughes et al. 2019; Price et al., 2019)… colonies that bleached and recovered (Ward et al., 2002).”

~ *This reads like regurgitated info. I recommend adding a concluding sentence to this paragraph that highlights what you want the reader to understand about the importance of your project from the inclusion of this paragraph in the intro.*

*~ There is differential bleaching observed dramatically across individuals so close to each other on the same reef. Has microsatellite work been done on these colonies? Is there a possibility of cryptic species/lineages across the bleaching susceptible and resistant corals here?*

*~ The authors talk a lot in the intro about how bleached individuals do not produce as many eggs as individuals that did not bleach for several coral species, but the authors findings (and included previous work on M. capitata) do not support this. Why include so many examples of this information in the intro then?*

“Reproductive effort of *M. capitata*, particularly oocyte characteristics and spawning, has shown little response to warming (Cox, 2007; Hagedorn et al., 2016).”

*~ This might be better to include in the discussion rather than the intro, since it makes some of the work you report here sound like it is not as novel.*

“This reproductive response may contribute to its ecological success along the fringing and patch reefs of Kāneʻohe Bay (Kolinski, 2004).”

*~ This statement is contradictory to some of the authors’ explanations of their results and statements in the discussion. I would consider including this association with different wording. Maybe say “This reproductive response may* ***have previously*** *contributed..” since you are talking about historical characteristics that may have been beneficial to the reproductive efforts of this species, but the authors are now highlighting how these characteristics may no longer be beneficial for some individuals in this paper.*

“This study was designed to assess selective processes in nature confronted by climate change while also exploring breeding techniques as an intervention strategy for coral restoration to maintain genetic diversity.”

~ *I like this statement that tries to summarize the importance of this study at the end of the intro, but the wording feels a bit confusing. Rework this sentence for more clarity.*

**Methods:**

**2.1 Selecting parent colonies and spawning events**

~ *The first paragraph of this section reads like it should be included in the introduction, instead of in the methods. Also, you highlight that Symbiodiniaceae symbionts are provided by the mothers to the eggs. Does this study assess dominant symbiont association of the mothers and/or larvae produced? That would help a lot with connecting impacts observed to more and less beneficial symbiont associations for this species.*

**2.3 Fertilization success and larval survivorship**

“Five days post-fertilization, 10-15 larvae per conical tank were placed in a 10-mL well-plate filled with 5-mL of FSW”

~ *Hyphens should be removed between 10 and mL, along with 5 and mL.*

**Results:**

**3.1 Sexual reproduction and egg traits**

“The proportion of colonies releasing gametes significantly differs by year (P < 0.001).

In 2017, the proportion of colonies participating in spawning events was 36% lower than in

2016.”

*~ It would help the reader to have the actual number of colonies that spawned for each group and each year listed here (or maybe in a table). Can also be in parentheses stated in a sentence.*

*~Also, this looks like some legacy effect of heat stress across B and NB since both had less spawning individuals in 2017. Would the findings for egg production and frequency of abnormal eggs not also represent possible legacy effects from the bleaching events? Might be an important consideration for how long it actually takes corals to fully “recover” from bleaching, whether or not they were visibly bleached.*

“In 2016, the spawning season following consecutive bleaching events, colonies that bleached and recovered had 22.5% higher mean total reproductive output than colonies that did not bleach, although this was not statistically significant”

*~ What explanation do the authors have for this since this seems contrary to bleached individuals being less fit than individuals that did not bleach.*

*~ Was any data collected to make sure the bleached and unbleached colonies were not in fact cryptic lineages/species? If not, then the incompatibility in BxNB and NBxB crosses could be a product of lineage crossing rather than the listed reasons by the authors. Indicating the authors might not be comparing apples to apples. This option should also be included in the manuscript to explain the differences in results if there was no testing of these colonies to eliminate this possibility.*

**3.2 Fertilization, survivorship, and settlement**

“There were no differences between BxB and the other three cross-types (P ≥ 0.342).”

*~ the font changes here, make sure this is formatted correctly*

Cell division was faster for within than between type crosses.

*~ Cryptic lineages could explain this as well.*

Egg donor’s bleaching history affects Percent swimming larvae and settlement of larvae. Along with lower survivorship of larvae from bleached donors. History of parent did not affect sperm.

BxB had highest mortality(~92%), then BxNB, and finally NBxNB (~77%) over 59 days.

*~Differences in egg quality? lipid class analyses of the eggs from B and NB would greatly benefit the impact and explanation of findings in this paper, due to the observed impacts of B and NB eggs on larvae survival. This would allow comparison of egg components, which might be driving differences in larval survival since eggs were of comparable volumes across B and NB groups and the results could be compared to previous measures of egg lipids from cited papers within this manuscript. If samples are available, the authors might consider adding these results.*

**Discussion:**

“These results demonstrate the impacts of marine heatwaves and coral bleaching on

reproductive capacity in the following spawning season with the capacity to recover in

subsequent nonbleaching years.”

~ *I agree with this statement by the authors, but I think it should also be emphasized here that similar impacts to egg production were observed* ***whether or not*** *the colonies were visibly bleached (B or NB) in this study. This supports that corals that are not bleached are also suffering some of the same impacts (i.e., the number of spawning individuals, number of eggs produced per bundle, number of abnormal eggs, etc.) as bleached corals after bleaching events, even if they are better positioned to survive and recover than bleached individuals.*

**4.1 Reproductive capacity after bleaching events:**

*~ Authors should define what is meant by “recover” here. Colonies after bleaching events are more susceptible to disease for more than a year after and can have reduced energy reserves for longer than that. If you consider these part of “recovery” of the coral, then ~5-6 months is not long enough to consider the corals as recovered. The authors also note in the intro that “The impacts of coral bleaching may last for months to years after the initial thermal stress” and cite a paper indicating 3 to 5 years for coral recovery, both of these statements contradict the statement made in the discussion of ~3 to 5 months for recovery. The authors need to be consistent in how they define “recovery” throughout the manuscript.*

“Maintaining egg traits such as size and biochemical composition would serve as an advantageous strategy to ensure ecological fitness of parents and their developing offspring (Moran & McAlister, 2009; Padilla-Gamiño et al., 2013; Jacobs & Podolsky, 2010). For example, there may be an optimal egg size that needs to be achieved to ensure successful fertilization (Levitan,1993; Levitan & Petersen, 1995). It is notable that the relationship between egg size and number of eggs per bundle in our study has shifted from prior studies; we found 10-12 eggs per bundle in 2016-2017 compared to 15-18 eggs per bundle in studies prior to 2010. (Cox, 2007; Padilla-Gamiño & Gates, 2012), and egg size was 11% larger in our study than previous studies. This apparent tradeoff in reproductive effort suggests plasticity in response to environmental changes and emphasizes the need for long-term studies to detect changes in sexual reproduction (Levitan et al., 2014; Hagedorn et al., 2016; Price et al., 2019; Schlesinger et al., 2019).”

~ *Why would eggs be larger than before? Authors should explain their thoughts on this finding more.*

*~ Also, this might be a good place to highlight that the plasticity utilized by B and NB may have been equally beneficial for B and NB before but that now egg quality might be suffering for B mothers due to continued impacts.*

**4.2 Parental effects on fertilization and offspring survivorship:**

“In this study, bleached colonies had similar fertilization success to nonbleached colonies, the thermally tolerant individuals will likely have a stronger selective advantage as warming continues to weed out thermally sensitive adults and their offspring (Drury et al., 2022).”

*~ I do not understand this statement, authors need to clarify more here. How can the B and NB colonies having similar fertilization success mean there is stronger selective advantage for NB adults and offspring? I understand why the authors are trying to link in natural selection towards more resilient individuals, but that is contrary to the results discussed in the beginning of this sentence. Especially since similar fertilization, number of eggs, and volume of eggs were found across both these groups for both years following the bleaching events. Rework this statement for clarity.*

“Beneficial cross-generational plasticity through maternal effects observed in offspring survivorship may be attributed to energetic provisioning through lipid reserves stored in the eggs and larvae (Jones et al., 2011; Padilla-Gamiño et al., 2013; Rivest et al., 2017), mitochondria (Dixon 15 et al., 2015), or vertical transmission of Symbiodinaceae from the parent into the eggs (Jones et al., 2010;Padilla-Gamiño et al., 2012; Quigley et al., 2016).”

~ *This statement should be fleshed out more and clarified further rather than just being a list of semi-unrelated traits. For example, what is meant by “Mitochondria” in this list because it does not look like it is connected to energy reserves.*

“M. capitata houses the endosymbionts Cladocopium spp. and Durusdinium spp., formerly Clade C and D, respectively.”

~ *It is outdated and improper to list genera levels of specific symbiont-host associations. Since only one coral species is assessed in the current study the specific symbiont species that associate with this coral in this area should be listed instead (i.e., Durusdinium trenchii instead of “Durusdinium spp.”)*

*~ Also, Padilla-Gamiño et al., 2013 does not actually identify the symbionts in M. capitata so it would be more correct to cite Padilla-Gamiño et al. 2012 in PLoSOne instead, or better yet de Souza et al. 2022 since that has more recent and specific symbiont identifications for M. capitata in Kāne‘ohe Bay.*

“In M. capitata, these symbionts are vertically transferred to the eggs creating offspring with different assemblages (Padilla-Gamiño et al., 2013) that could confer different physiological attributes to the offspring.”

*~ This should also be Padilla-Gamiño et al., 2012 PLoSOne that looked at symbionts in eggs, rather than 2013.*

“For example, Little et al. (2004) found that Acropora juveniles grew faster when infected with clade C than clade D, and Abrego et al. (2008)”

*~ Same comment about symbiont names here. Look up the specific genera for these symbiont-host associations. It is not appropriate to call the symbionts by the antiquated “Clades” designations, also the authors should be consistent with how they reference the symbionts throughout the manuscript rather than changing between genera names and clade names.*

“Our results suggest that nonbleached colonies have higher ecological fitness than their bleached counterparts and may benefit the maintenance of genetic diversity while guiding populations towards higher thermal tolerance in a warming ocean.”

*~ Authors discuss the importance of the photosynthetic endosymbiont to the host and that this symbiont is vertically transmitted to the larvae by the mother. After disturbance the dominant Symbiodiniaceae community members can shift rapidly back to a previous non-disturbed community state (sometimes within a few months to a year, see references below this comment). It is important to know what symbionts were at play in B and NB parents (and the sampled larvae) before making any statements about what impacts these may have had on the larvae survivorship in the current study.*

***Were the symbionts ever identified for the parent colonies or subset of larvae to determine if any claims in this paragraph were relevant to the corals and findings of this study?*** *If not, then the concluding sentence of this paragraph should be removed since no data collected by the authors corroborates the dominant symbiont of the mothers or larvae.*

- Elder H, Million WC, Bartels E, Krediet CJ, Muller EM, Kenkel CD (2022) Long-term maintenance of a heterologous symbiont association in *Acropora palmata* on natural reefs. The ISME Journal
- Thornhill DJ, LaJeunesse TC, Kemp DW, Fitt WK, Schmidt GW (2006) Multi-year, seasonal genotypic surveys of coral-algal symbioses reveal prevalent stability or post-bleaching reversion. Marine Biology 148:711-722

**4.3 Interventions for thermal tolerance:**

“manipulation of coral-algal endosymbiosis”

*~ the authors have not referenced the Symbiodiniaceae as “algae” yet in this manuscript or defined the use of “algae” to mean Symbiodiniaceae. For consistency in text and lack of confusion with readers this should be changed to reflect the terms used to reference the Symbiodiniaceae symbionts throughout the manuscript.*

“Human interventions, such as applying selective breeding in coral sexual propagation, has been proposed as one of the viable options to maintain genetic diversity and increase resilience in restoration efforts (Barott et al., 2021; Epstein et al., 2003; van Oppen et al., 2015, 2017; NASEM, 2019; Hancock et al., 2021); however, feasibility to potentially scale up efforts remain limited and costly without full understanding of tradeoffs (Edwards et al., 2015; Chamberland et al., 2017).”

*~ Need commas here*

*~ The way this is written it sounds here like only thermally resistant colonies (NB colonies in this paper) should be considered for selective breeding, but thermal resilience is not the only trait corals need to survive anthropogenic climate change, which also causes increased disease prevalence and ocean acidification along with other factors. Since B adults have also been successful thus far, it is likely they have a different trait necessary for acclimating well to their home reefs, like disease resistance, greater trophic plasticity, or that they utilize different trade-offs to deal with thermal stress. The data here does not necessarily support selective breeding for thermal tolerance with individuals that do not bleach, but instead the preference for using eggs from NB parents when creating crosses following bleaching events. Therefore, the authors should consider making less definitive statements here.*

**Figures:**

Figure 3: *Reproductive output was higher in bleached colonies and had fewer abnormalities than NB colonies, how do the authors explain this in relation to their statements that the bleached colonies are less fit in the discussion?*

Figure 4: *Panel B should include x-axis labels for clarity. It is confusing to look at a separate panel for the axis labels, especially since only those two panels in the figure share labels.*
