# Supplementary material for: Parental effects provide an opportunity for coral resilience following major bleaching events
Source: PLoS One. 2025 Jan 7;20(1):e0290479. doi: 10.1371/journal.pone.0290479 (PMC11706449; doi:10.1371/journal.pone.0290479)
Supplement: Attachment — Submitted filename: PLOSONE_Review.docx [file pone.0290479.s002.docx]

**PLOS ONE Review**

**General comments**

Lenz and colleagues present results from experiments spanning multiple years to investigate the potential for multigenerational effects of thermal stress on the coral *Montipora capitata.* They found that while thermal stress impaired aspects of reproduction regardless of if a colony bleached or not, parental bleaching history was an important driver of offspring success. These findings supporting the concept that bleaching-resistant colonies could be targeted for selective breeding to produce offspring that to support the restoration of more thermally-tolerant coral populations. The authors do an excellent job of highlighting the importance of their findings and avoid overextending their conclusions beyond the scope of their study. Their findings are particularly timely given the increasing severity, prevalence, and mortality caused by thermal stress on coral reefs across the globe. I have relatively minor comments that are articulated below that I recommend addressing before the ms is published.

There are no line numbers on the submission, thus I have not included them below but did my best to describe where in the ms I am referring to by referencing paragraphs within sections.

**Introduction**: The introduction is well written and provides a thorough yet concise overview of how thermal stress can impact coral reproduction and population dynamics, while also highlighting the knowledge gap this paper addresses (intra- and intergenerational impacts of bleaching).

Paragraph (henceforth, P) 4: The sentence regarding high recruitment in Mo’orea after the 2016 thermal stress is incomplete/lacking details compared to the other examples. The examples presented in P3 and P4 provide clear links between thermal stress/bleaching and reproduction in corals. However, the Mo’orea example suggests that the minor 2016 bleaching event may have had the potential to affect reproductive output, but provides no concrete information that some corals were reproductively compromised. How it is currently written, one could conclude that the minor bleaching in 2016 did not impact reproduction. Suggest revising to better leverage this example for the introduction.

P5: Sentence with “~70% of reef corals on shallow reefs” – is there a reason that these corals are distinguished as reef corals? If not, remove to avoid confusion, or if there is an important distinction between reef corals and other corals, provide details for clarity.

**Methods**

P2: Do the authors have any bleaching history of the colonies used in this study during 2014? If so, that would be worthwhile to include here or as supplemental information.

P5: For quantification of larval survivorship – was this recorded as the number of larvae remaining in an individual well-plate, or did this take into account the number of settlers (which is needed to quantify larval survivorship)? For example, if a well-plate started with 10 larvae, and 5 had settled after 7 days and 5 remained swimming, was this recorded as 100% survivorship (5 out of the 5 larvae remaining) or 50% survivorship (5 of the initial 10 placed in the well-plate)? This should be clarified in the methods section.

P6 (second sentence) – revise for clarity. Mentions using a generalized mixed effects model twice in the same sentence, but is only referring to one model.

P7: The survivorship curve analysis includes days 23 and 27, but these days are not mentioned in the previous section regarding when the number of offspring alive were counted (days 6, 7, 28, 53, and 59).

P7: Was the effect of fragmentation included in any of the models to test if fragmenting impacted reproduction? Given the reported size of the fragments, there is not reason to believe there would be any impact, but this would be worth mentioning somewhere in the ms.

**Results**

**Discussion:** The discussion is well written and thorough. The authors did a nice job of placing their findings in the context of previous work (e.g. reduction in no. of eggs/bundle), proposing potential hypotheses for the patterns they observed (and supporting evidence), expanding the discussion to broader concepts of population ecology, and explaining how these results can be used to inform interventions like selective breeding.

**Figures**

Figure 2: Suggest choosing a color palette that facilitates distinguishing between B and NB colonies. I can understand why these were chosen to look like healthy (NB) vs. bleached (B) colonies, but the colors are difficult to quickly distinguish.

Figure 3: 3A -Lines are not appropriate for this type of plot – these data would be more clearly presented as a bar plot since each night of spawning is a discrete event, not continuous as the lines connecting the dots imply.

3B-E: suggest adding a legend for the B and NB colors even though it is on the x-axis labels (and hidden in the 2016 panel of Figure 3A.

Figure 4B: Add x-axis labels to this plot, it is difficult to follow them up from Fig 4C.

4C: Error bars on stacked bar plots make for very busy figures that are difficult to interpret and take away from the data being shown. Suggest individual bars for each of the developmental stages for each cross – this would make the difference in developmental stages easily observable by looking at Fig 4C.

4D-F: Similar to Fig 4, these colors are similar and thus hard to distinguish, especially given the that these are small panels within a 6-panel figure. Suggest selecting a different color scheme.
