# Supplementary material for: Parental effects provide an opportunity for coral resilience following major bleaching events
Source: PLoS One. 2025 Jan 7;20(1):e0290479. doi: 10.1371/journal.pone.0290479 (PMC11706449; doi:10.1371/journal.pone.0290479)
Supplement: Attachment — Submitted filename: 4. Response to Reviewers Final.docx [file pone.0290479.s003.docx]

**Journal requirements:**

When submitting your revision, we need you to address these additional requirements.

1. Please ensure that your manuscript meets PLOS ONE's style requirements, including those for file naming. The PLOS ONE style templates can be found at

[https://journals.plos.org/plosone/s/file?id=wjVg/PLOSOne_formatting_sample_main_body.pdf](https://urldefense.com/v3/__https:/journals.plos.org/plosone/s/file?id=wjVg*PLOSOne_formatting_sample_main_body.pdf__;Lw!!PvDODwlR4mBZyAb0!XuwFPj6fQDP7P6rzVi83CuVZHDP9fD2L2rF2-lSF_Y6KpeH5aJ0aUResU0mtRYL4UzKs92eWIByYUp_6$) and

[https://journals.plos.org/plosone/s/file?id=ba62/PLOSOne_formatting_sample_title_authors_affiliations.pdf](https://urldefense.com/v3/__https:/journals.plos.org/plosone/s/file?id=ba62*PLOSOne_formatting_sample_title_authors_affiliations.pdf__;Lw!!PvDODwlR4mBZyAb0!XuwFPj6fQDP7P6rzVi83CuVZHDP9fD2L2rF2-lSF_Y6KpeH5aJ0aUResU0mtRYL4UzKs92eWID-ZDGPq$)

**Response 1: We have ensured that the submitted revisions follow the PlosONE style requirements and file naming as advised by the links provided above.**

2. Thank you for stating the following financial disclosure:

“National Science Foundation Graduate Research Fellowship Program

Paul G. Allen Family Foundation.”

Please state what role the funders took in the study. If the funders had no role, please state: "The funders had no role in study design, data collection and analysis, decision to publish, or preparation of the manuscript."

If this statement is not correct you must amend it as needed.

Please include this amended Role of Funder statement in your cover letter; we will change the online submission form on your behalf.

**Response 2: The funders had no role in the design, execution, data collection, analysis decision to publish or preparation of the manuscript and will amend with the recommended language to include in our Funding Statement. See Response 3 below.**

If the funders had no role, please state: "The funders had no role in study design, data collection and analysis, decision to publish, or preparation of the manuscript."

3. Thank you for stating the following in the Acknowledgments Section of your manuscript:

“This work was supported by funding from Paul G. Allen Family Foundation to RG and the National Science Foundation Graduate Research Fellowships to EAL. We would like to thank members of the Gates Coral Laboratory for their technical support and advice, especially Jen Davidson and Dr. James Guest, we are grateful to Mary Hagedorn, Amy Moran, and Peter Marko for their feedback on the manuscript, the many volunteers especially Dyson Chee, Megan Buras, Katie Allen, Shayne Fabian, and Kat McPherson and the security Greg Miranda and Moses at Moku o Loʻe who ensured safety and the success of this research. We dedicate this research to Dr. Ruth D. Gates and her infectious enthusiasm that pushed human-assisted evolution to the forefront of coral biology – you rock!”

We note that you have provided funding information that is currently declared in your Funding Statement. However, funding information should not appear in the Acknowledgments section or other areas of your manuscript. We will only publish funding information present in the Funding Statement section of the online submission form.

Please remove any funding-related text from the manuscript and let us know how you would like to update your Funding Statement. Currently, your Funding Statement reads as follows:

“National Science Foundation Graduate Research Fellowship Program

Paul G. Allen Family Foundation.”

Please include your amended statements within your cover letter; we will change the online submission form on your behalf of the coauthors:

**Response 3: Thank you for the clarification, we have removed information regarding our funders from the acknowledgements and would like to include the following as our Funding Statement:**

**“This work was supported by funding from Paul G. Allen Family Foundation to RDG and the National Science Foundation Graduate Research Fellowships to EAL. The funders had no role in study design, data collection and analysis, decision to publish, or preparation of the manuscript.”**

4. We note that you have stated that you will provide repository information for your data at acceptance. Should your manuscript be accepted for publication, we will hold it until you provide the relevant accession numbers or DOIs necessary to access your data. If you wish to make changes to your Data Availability statement, please describe these changes in your cover letter and we will update your Data Availability statement to reflect the information you provide.

**Response 4: Data repository is on GitHub**

[**https://github.com/ealenz/Mcap-BNB-Reproduction-SelectiveBreeding**](https://github.com/ealenz/Mcap-BNB-Reproduction-SelectiveBreeding)

5. Please include your full ethics statement in the ‘Methods’ section of your manuscript file. In your statement, please include the full name of the IRB or ethics committee who approved or waived your study, as well as whether or not you obtained informed written or verbal consent. If consent was waived for your study, please include this information in your statement as well.

**Response 5: The study did not require an IRB or ethics committee and this information is not relevant to the research that was conducted.**

6. We note that Figures 1B, 2, and 4A in your submission contain copyrighted images. All PLOS content is published under the Creative Commons Attribution License (CC BY 4.0), which means that the manuscript, images, and Supporting Information files will be freely available online, and any third party is permitted to access, download, copy, distribute, and use these materials in any way, even commercially, with proper attribution. For more information, see our copyright guidelines: [http://journals.plos.org/plosone/s/licenses-and-copyright](https://urldefense.com/v3/__http:/journals.plos.org/plosone/s/licenses-and-copyright__;!!PvDODwlR4mBZyAb0!XuwFPj6fQDP7P6rzVi83CuVZHDP9fD2L2rF2-lSF_Y6KpeH5aJ0aUResU0mtRYL4UzKs92eWINpaNP1x$).

We require you to either (1) present written permission from the copyright holder to publish these figures specifically under the CC BY 4.0 license, or (2) remove the figures from your submission:

1. You may seek permission from the original copyright holder of Figures 1B, 2, and 4A to publish the content specifically under the CC BY 4.0 license.

We recommend that you contact the original copyright holder with the Content Permission Form ([http://journals.plos.org/plosone/s/file?id=7c09/content-permission-form.pdf](https://urldefense.com/v3/__http:/journals.plos.org/plosone/s/file?id=7c09*content-permission-form.pdf__;Lw!!PvDODwlR4mBZyAb0!XuwFPj6fQDP7P6rzVi83CuVZHDP9fD2L2rF2-lSF_Y6KpeH5aJ0aUResU0mtRYL4UzKs92eWIAPURq35$)) and the following text:

“I request permission for the open-access journal PLOS ONE to publish XXX under the Creative Commons Attribution License (CCAL) CC BY 4.0 ([http://creativecommons.org/licenses/by/4.0/](https://urldefense.com/v3/__http:/creativecommons.org/licenses/by/4.0/__;!!PvDODwlR4mBZyAb0!XuwFPj6fQDP7P6rzVi83CuVZHDP9fD2L2rF2-lSF_Y6KpeH5aJ0aUResU0mtRYL4UzKs92eWINTZspXO$)). Please be aware that this license allows unrestricted use and distribution, even commercially, by third parties. Please reply and provide explicit written permission to publish XXX under a CC BY license and complete the attached form.”

Please upload the completed Content Permission Form or other proof of granted permissions as an "Other" file with your submission.

In the figure caption of the copyrighted figure, please include the following text: “Reprinted from [ref] under a CC BY license, with permission from [name of publisher], original copyright [original copyright year].”

2. If you are unable to obtain permission from the original copyright holder to publish these figures under the CC BY 4.0 license or if the copyright holder’s requirements are incompatible with the CC BY 4.0 license, please either i) remove the figure or ii) supply a replacement figure that complies with the CC BY 4.0 license. Please check copyright information on all replacement figures and update the figure caption with source information. If applicable, please specify in the figure caption text when a figure is similar but not identical to the original image and is therefore for illustrative purposes only.

**Response 6: These images were produced and provided by the lead author of the paper. I, Elizabeth Lenz, give permission for these images to be used for the publication.**

7. Please include your tables as part of your main manuscript and remove the individual files. Please note that supplementary tables should be uploaded as separate "supporting information" files.

**Response 7: Thank you, we have included the tables as part of the main manuscript.**

8. Please review your reference list to ensure that it is complete and correct. If you have cited papers that have been retracted, please include the rationale for doing so in the manuscript text, or remove these references and replace them with relevant current references. Any changes to the reference list should be mentioned in the rebuttal letter that accompanies your revised manuscript. If you need to cite a retracted article, indicate the article’s retracted status in the References list and also include a citation and full reference for the retraction notice.

**Response 8: We reviewed our reference list and ensured it is updated.**

Additional Editor Comments:

Message from the Editorial Office regarding a deceased co-author: For a deceased co-author, this would need to be indicated in the title page of the manuscript in addition to the acknowledgments. When submitting your manuscript, as the corresponding author you must accept the responsibility for the integrity of all data collected and analyzed by the deceased author. Suggested text for this in the Acknowledgements section is "[Author] passed away before the submission of the final version of this manuscript. [Corresponding author] accepts responsibility for the integrity and validity of the data collected and analyzed." Please also add a "†" symbol next to the author's name in the author list and include a note that this author is deceased.

**Response 9: and have included the following:
“Dr. Ruth D. Gates passed away before the submission of the final version of this manuscript. Dr. Elizabeth A. Lenz accepts responsibility for the integrity and validity of the data collected and analyzed."**

**Please also add a "†" symbol next to the author's name in the author list and include a note that this author is deceased.**

[Note: HTML markup is below. Please do not edit.]

**Reviewers' comments:

Reviewer's Responses to Questions**

**Comments to the Author**

*1. Is the manuscript technically sound, and does the data support the conclusions?

The manuscript must describe a technically sound piece of scientific research with data that supports the conclusions. Experiments must have been conducted rigorously, with appropriate controls, replication, and sample sizes. The conclusions must be drawn appropriately based on the data presented.*

Reviewer #1: Partly

Reviewer #2: Yes

Reviewer #3: Partly

**Response to Reviewers: We appreciate the honest response by the reviewers and hope we have fully addressed the concerns.**

*2. Has the statistical analysis been performed appropriately and rigorously?*

Reviewer #1: Yes

Reviewer #2: Yes

Reviewer #3: Yes

**Response to Reviewers: We appreciate the positive response by the reviewers and appreciate the acknowledgement of our work in our statistical analyses of the study.**

*3. Have the authors made all data underlying the findings in their manuscript fully available?

The* [*PLOS Data policy*](https://urldefense.com/v3/__http:/www.plosone.org/static/policies.action*sharing__;Iw!!PvDODwlR4mBZyAb0!XuwFPj6fQDP7P6rzVi83CuVZHDP9fD2L2rF2-lSF_Y6KpeH5aJ0aUResU0mtRYL4UzKs92eWILPwK2on$) *requires authors to make all data underlying the findings described in their manuscript fully available without restriction, with rare exception (please refer to the Data Availability Statement in the manuscript PDF file). The data should be provided as part of the manuscript or its supporting information, or deposited to a public repository. For example, in addition to summary statistics, the data points behind means, medians and variance measures should be available. If there are restrictions on publicly sharing data—e.g. participant privacy or use of data from a third party—those must be specified.*

Reviewer #1: Yes

Reviewer #2: Yes

Reviewer #3: Yes

**Response: We appreciate the positive responses from the reviewers in meeting the PLOS data policies.**

*4. Is the manuscript presented in an intelligible fashion and written in standard English?

PLOS ONE does not copyedit accepted manuscripts, so the language in submitted articles must be clear, correct, and unambiguous. Any typographical or grammatical errors should be corrected at revision, so please note any specific errors here.*

Reviewer #1: Yes

Reviewer #2: Yes

Reviewer #3: Yes

**Response: We are grateful to the reviewers for taking the time to copy edit the manuscript and ensure the contents meets the PLOS ONE requirements.**

*5. Review Comments to the Author

Please use the space provided to explain your answers to the questions above. You may also include additional comments for the author, including concerns about dual publication, research ethics, or publication ethics. (Please upload your review as an attachment if it exceeds 20,000 characters)*

Reviewer #1: Reviewer comments on lines following "~".
Direct quotes being addressed from manuscript are in "".
Review comments are broken up by sections and sub-headers in sections.

**Response: Thank you for providing clear instructions and guidance on the feedback provided. We greatly appreciate the clarity and thoughtfulness behind the comments.**

Introduction:

“Coral bleaching is known to impact sexual reproduction (Baird & Marshall, 2002; Fisch et al.,2019) and recruitment (Hughes et al. 2019; Price et al., 2019)… colonies that bleached and recovered (Ward et al., 2002).”
~ This reads like regurgitated info. I recommend adding a concluding sentence to this paragraph that highlights what you want the reader to understand about the importance of your project from the inclusion of this paragraph in the intro.

**Response: We appreciate the feedback on providing a stronger concluding sentence to help contextualize the study for the readers and provide stronger understanding.**

**LN 81-93: “Previous studies have identified some of the way coral bleaching can impact aspects of sexual reproduction** [**(Baird & Marshall, 2002; Fisch et al., 2019)**](https://paperpile.com/c/pW0cc0/zTMW4+hgzVo) **and dampen recruitment** [**(Hughes et al.**](https://paperpile.com/c/pW0cc0/KrDvb) **2019; Price et al., 2019). For example, after the 1987 coral bleaching event in the Caribbean, *Orbicella annularis* recovered from bleaching by metabolizing tissue biomass, but did not complete gametogenesis in the following months, whereas colonies that had not bleached of the same species were able to develop and release gametes** [**(Szmant & Gassman, 1990)**](https://paperpile.com/c/pW0cc0/QP05a)**. Similarly, during the 1998 bleaching event on the Great Barrier Reef, bleached corals showed high variation in reproduction compared to colonies resistant to bleaching nearby that experienced the same thermal stress. For acroporid species, reproductive polyps were more common in colonies that did not bleach, with larger eggs at higher densities per polyp than colonies that bleached and recovered (Ward et al., 2002). More resolution is needed to better understand the impact and extent of coral bleaching events on the early life cycles of coral, from the stress event through recruitment.”**

~ There is differential bleaching observed dramatically across individuals so close to each other on the same reef. Has microsatellite work been done on these colonies? Is there a possibility of cryptic species/lineages across the bleaching susceptible and resistant corals here?

**Response: We did not conduct microsatellites on these colonies and cannot conclude a pattern of cryptic species/lineages within the samples. However, there is evidence that colonies more vulnerable to bleaching have clade C symbionts and that corals more resistant to bleaching are dominated by thermotolerant clade D (Cunning et al. 2016). Since 2014, former members of the Gates Coral Lab, including Drs. Raphael Ritson Williams, Christopher Wall, Hollie Putnman, Ross Cunning, Shayle Matsuda, Katie Barott and now the Coral Resilience Lab at the Hawaiʻi Institute of Marine Biology continue to conduct studies to understand and explain the mechanisms that drives the differential bleaching responses to marine heatwaves in Kāneʻohe Bay. This mechanism behind this differential bleaching phenomenon remains unknown.**

~ The authors talk a lot in the intro about how bleached individuals do not produce as many eggs as individuals that did not bleach for several coral species, but the authors findings (and included previous work on M. capitata) do not support this. Why include so many examples of this information in the intro then? Maybe just say concisely in discussion.

**Response: The intent of the introduction was to provide a thorough overview of what has been observed in the past, particularly with regards to corals in the acroporidae family. We believe it is worthwhile to keep as is and that a hypothesis for the difference in results could be attributed to acclimation over the last decade as waters have warmed.**

“This reproductive response may contribute to its ecological success along the fringing and patch reefs of Kāneʻohe Bay (Kolinski, 2004).”
~ This statement is contradictory to some of the authors’ explanations of their results and statements in the discussion. I would consider including this association with different wording. Maybe say “This reproductive response may have previously contributed..” since you are talking about historical characteristics that may have been beneficial to the reproductive efforts of this species, but the authors are now highlighting how these characteristics may no longer be beneficial for some individuals in this paper.

**Response: We thank the reviewer for this suggestion and have revised the sentence to state the following:**

**LN 135-137 “This reproductive response may have contributed to its ecological success along the fringing and patch reefs of Kāneʻohe Bay in the past (Kolinski, 2004).”**

“This study was designed to assess selective processes in nature confronted by climate change while also exploring breeding techniques as an intervention strategy for coral restoration to maintain genetic diversity.”
~ I like this statement that tries to summarize the importance of this study at the end of the intro, but the wording is a bit confusing. Rework this sentence for more clarity.

**Response: We have revised the concluding sentence of the introduction:
LN 157-160 “This study was designed to assess the impacts of consecutive bleaching events on the early stages within the coral life cycle and selective processes already occurring in nature while also testing basic breeding techniques as an intervention strategy for coral restoration to maintain genetic diversity and promote resilience.”**

Methods:

2.1 Selecting parent colonies and spawning events

~ The first paragraph of this section reads like it should be included in the introduction, instead of in the methods. Also, you highlight that Symbiodiniaceae symbionts are provided by the mothers to the eggs. Does this study assess dominant symbiont association of the mothers and/or larvae produced? That would help a lot with connecting impacts observed to more and less beneficial symbiont associations for this species.

**Response: While we agree that some of this information that describes the early life cycle of *M. capitata* could be placed in the introduction we have decided to keep the placement as is in the methods as this information is the driver for how the study was conducted. Based on feedback from multiple readers, this placement makes it easier for readers to follow along.**

2.3 Fertilization success and larval survivorship

“Five days post-fertilization, 10-15 larvae per conical tank were placed in a 10-mL well-plate filled with 5-mL of FSW”
~ Hyphens should be removed between 10 and mL, along with 5 and mL.

**Response: All hyphens have been removed from the manuscript where requested. We thank the reviewer for identifying that error.**

Results:

3.1 Sexual reproduction and egg traits

“The proportion of colonies releasing gametes significantly differs by year (P < 0.001). In 2017, the proportion of colonies participating in spawning events was 36% lower than in 2016.”
~ It would help the reader to have the actual number of colonies that spawned for each group and each year listed here (or maybe in a table). Can also be in parentheses stated in a sentence.

**Response: We appreciate the reviewer request for clarity on the number of total colonies that spawned. All 15 parental colonies that bleached (and recovered) and did not bleach spawned at least once during each spawning season in 2016 and 2017, however the number of times the colonies spawned during the season was lower in 2017 compared to 2016. For instance, parental colonies that had bleached and recovered spawned between 5 to 9 times in 2016 and only 1 to 7 times in 2017. For nonbleached parental colonies, they spawned 5 to 11 times in 2016 and only 3 to 7 times in 2017. We revised Figure 3A to hopefully better show the proportion of colonies that spawned each night of the two spawning seasons.**

~Also, this looks like some legacy effect of heat stress across B and NB since both had less spawning individuals in 2017. Would the findings for egg production and frequency of abnormal eggs not also represent possible legacy effects from the bleaching events? Might be an important consideration for how long it actually takes corals to fully “recover” from bleaching, whether or not they were visibly bleached.

**Response: Yes, there were fewer parental colonies that spawned in the second spawning season of this study, and it is likely that egg development and abnormality are impacted by the legacy of multiple bleaching events. We know that bleached corals can take approximately 3 months to recover biomass and chlorophyll levels (Wall et al. 2019), and this recovery period could affect gametogenesis, which lasts 8 months. We address that there may be selective effects in maintaining the mean reproductive output and egg volume in both seasons. A recent publication by Brown et al. 2023 in PNAS does provide a more thorough physiological understanding of the impacts of heatwaves on bleached and nonbleached pairs in Kāneʻohe Bay. The results of this study which provides an understanding of invisible stress on the nonbleached *Montipora capitata* colonies are included in the discussion.**

**LN 350-368: “*M. capitata* appears to maintain reproductive resilience after consecutive marine heatwaves and coral bleaching events, as evidenced by continuing synchronous broadcast spawning and production of viable eggs and sperm. These results are consistent with prior studies examining the influence of environmental and biological factors on *M. capitata*gametogenesis and spawning in Kāneʻohe Bay** [**(Cox, 2007; Padilla-Gamiño**](https://paperpile.com/c/pW0cc0/7nvN+bj0br+uIfh) **et al*.***[**, 2014)**](https://paperpile.com/c/pW0cc0/7nvN+bj0br+uIfh)**. For instance, Padilla-Gamiño et al. (2014) found similar rates of gametogenesis along a strong sedimentation gradient. Further, Cox (2007) found no differences in reproductive output, eggs per bundle, and egg size between B and NB parents in the spawning season immediately following the 2004 mild warming event. Resilience in *M. capitata* may be due to its capacity to maintain energetic stability under stress** [**(Wall**](https://paperpile.com/c/pW0cc0/VfCxM) **et al., 2019**[**)**](https://paperpile.com/c/pW0cc0/VfCxM)**, here evident by the completion of gametogenesis even at the cost of producing fewer eggs per bundle with higher proportion of irregularity in shape in 2016 than in 2017. One hypothesis to explain similar reproductive traits in bleached and nonbleached parents, is that after the thermal stress (Sept-Oct), there is still time for the colonies to recover (~5-6 months) and develop gametes that can be released during the spawning season (May-August) (Padilla-Gamiño et al. 2014, Wall et al. 2019). Furthermore, Rodrigues & Padilla-Gamino (2022) found that M. capitata colonies that bleached allocated 10% more carbon to gametes despite bleaching by limiting the allocation of carbon to adult tissues, with 50–80% less carbon allocated to bleached compared to non‑bleached adult colonies. Compared to other species, M. capitata prioritizes gametogenesis at the expense of the adult colony.”**

“In 2016, the spawning season following consecutive bleaching events, colonies that bleached and recovered had 22.5% higher mean total reproductive output than colonies that did not bleach, although this was not statistically significant”
~ What explanation do the authors have for this since this seems contrary to bleached individuals being less fit than individuals that did not bleach.

**Response: While we did observe a difference in the mean, the results are not significant. One possibility is that this is a stress response where colonies produced more egg-sperm bundles to increase the potential contribution to the gene pool.**

**We have included in LN 364-368:**

**“Furthermore, Rodrigues & Padilla-Gamino (2022) found that *M. capitata* colonies that bleached allocated 10% more carbon to gametes despite bleaching by limiting the allocation of carbon to adult tissues, with 50–80% less carbon allocated to bleached compared to non‑bleached colonies. Compared to other species, M. capitata prioritizes gametogenesis at the expense of the adult colony.”**

~ Was any data collected to make sure the bleached and unbleached colonies were not in fact cryptic lineages/species? If not, then the incompatibility in BxNB and NBxB crosses could be a product of lineage crossing rather than the listed reasons by the authors. Indicating the authors might not be comparing apples to apples. This option should also be included in the manuscript to explain the differences in results if there was no testing of these colonies to eliminate this possibility.

**Response: Preliminary microsatellites were not done on the studied colonies to prove these individuals were not genetic clones of the other. We have included a sentence in the discussion to highlight that this is a limitation of our study.**

**LN 411-416 “The lack of compatibility observed in crosses between B × NB and B × NB could potentially result from lineage crossing. However, in our study, we were unable to analyze the genetic composition of the parent organisms and we couldn't determine if they belonged to distinct parental lineages. Future studies should take into account parental lineages to better understand gamete compatibility, inheritance patterns and traits that can lead to increased genetic diversity or novel offspring phenotypes.”**

3.2 Fertilization, survivorship, and settlement

“There were no differences between BxB and the other three cross-types (P ≥ 0.342).”
~ the font changes here, make sure this is formatted correctly

**Response: Thank you, we have made this correction.**

Cell division was faster for within than between type crosses.
~ Cryptic lineages could explain this as well.

**Response: We were unable to determine this given the extent of this study and encourage others pursuing similar work in coral sexual reproduction to further investigate cryptic lineages. The original question behind the study was not designed to address cryptic lineages unfortunately so this remains unanswered and we acknowledge this in the discussion. We have included a sentence in the discussion to highlight that this is a limitation of our study (see above as well).**

**LN 411-416 “The lack of compatibility observed in crosses between B × NB and B × NB could potentially result from lineage crossing. However, in our study, we were unable to analyze the genetic composition of the parent organisms and we couldn't determine if they belonged to distinct parental lineages. Future studies should take into account parental lineages to better understand gamete compatibility, inheritance patterns and traits that can lead to increased genetic diversity or novel offspring phenotypes.”**

Egg donor’s bleaching history affects Percent swimming larvae and settlement of larvae. Along with lower survivorship of larvae from bleached donors. History of parent did not affect sperm. BxB had highest mortality(~92%), then BxNB, and finally NBxNB (~77%) over 59 days.

~Differences in egg quality? lipid class analyses of the eggs from B and NB would greatly benefit the impact and explanation of findings in this paper, due to the observed impacts of B and NB eggs on larvae survival. This would allow comparison of egg components, which might be driving differences in larval survival since eggs were of comparable volumes across B and NB groups and the results could be compared to previous measures of egg lipids from cited papers within this manuscript. If samples are available, the authors might consider adding these results.

**Response:**

**We agree that looking at the total lipid content and composition of lipids in the eggs would help explain the results in larval survivorship, unfortunately given the amount of gamete material available to conduct this study, we were unable to pursue as the priority of the question was to compare early life stages to recruitment.**

**Padilla-Gamiño et al. (2013) showed that total lipid levels, wax esters and triacylglycerols in the eggs did not change even though parents lived in different environments and had different lipid levels. Thus, eggs released by corals that are acclimatized/adapted to different environments are provisioned similarly in terms of stored lipids. To date, we do not know how lipids change in eggs from bleaching colonies of *M. capitata*.**

**We appreciate the feedback and unfortunately do not have any materials to test this and encourage future work to scale up the number of colonies and samples collected to increase resolution on how parental effects are influenced.**

Discussion:

“These results demonstrate the impacts of marine heatwaves and coral bleaching on reproductive capacity in the following spawning season with the capacity to recover in subsequent nonbleaching years.”
~ I agree with this statement by the authors, but I think it should also be emphasized here that similar impacts to egg production were observed whether or not the colonies were visibly bleached (B or NB) in this study. This supports that corals that are not bleached are also suffering some of the same impacts (i.e., the number of spawning individuals, number of eggs produced per bundle, number of abnormal eggs, etc.) as bleached corals after bleaching events, even if they are better positioned to survive and recover than bleached individuals.

**Response: The first paragraph of the discussion has been revised to state:
LN 336-346: “The unprecedented, consecutive warming events in 2014 and 2015 in Kāneʻohe Bay, Hawaiʻi influenced the reproductive capacity of *M. capitata*. In the first spawning season following the 2015 bleaching event, *M. capitata* had eggs with similar volume but fewer eggs were packaged within bundles. In 2016, egg abnormality was higher than in 2017 regardless of parental bleaching history. These results demonstrate the influence of marine heatwaves on parental effects and reproductive capacity in the following spawning season. Parental colonies that bleached and did not bleach had similar reproductive output, number of eggs per bundle and egg abnormality. However, delayed beneficial maternal effects were observed in offspring from parents resistant to bleaching. These results demonstrate that although *M. capitata* has the energetic capacity to continue reproduction despite bleaching response, cross-generational impacts occur (Byrne et al., 2020), with possible ecological consequences downstream.”**

4.1 Reproductive capacity after bleaching events:

~ Authors should define what is meant by “recover” here. Colonies after bleaching events are more susceptible to disease for more than a year after and can have reduced energy reserves for longer than that. If you consider these part of “recovery” of the coral, then ~5-6 months is not long enough to consider the corals as recovered. The authors also note in the intro that “The impacts of coral bleaching may last for months to years after the initial thermal stress” and cite a paper indicating 3 to 5 years for coral recovery, both of these statements contradict the statement made in the discussion of ~3 to 5 months for recovery. The authors need to be consistent in how they define “recovery” throughout the manuscript.

**Response: We have revised the manuscript to specify that recovery is defined in this study as the return of algal symbionts and regain its original pigmentation/coloration. We know that bleached corals can take around 3 months to recover biomass and chlorophyll levels (Wall et al. 2019).**

**LN 122-127 “During both events in Kāneʻohe Bay, colonies of the dominant reef-building coral, *Montipora capitata*, visibly bleached or remained pigmented during prolonged heat stress (Figure 1B). Despite widespread bleaching, approximately 70% of *M. capitata* that bleached in 2014 and 2015 were considered recovered by the following December and January based on visual coloration (Bahr et al, 2018; Cunning et al., 2016; Wall et al. 2019; Matsuda et al., 2020; Barott et al., 2021; Brown et al., 2023).”**

“Maintaining egg traits such as size and biochemical composition would serve as an advantageous strategy... long-term studies to detect changes in sexual reproduction (Levitan et al., 2014; Hagedorn et al., 2016; Price et al., 2019; Schlesinger et al., 2019).”
~ Why would eggs be larger than before? Authors should explain their thoughts on this finding more.

**Response: We have included the following LN 380-384 “While further examination of egg traits, such as total lipid content and composition of lipid classes, was beyond the scope of this study due to limited material available, larger egg volume could be beneficial in storing lipids and carbohydrates as well as increased surface area for slower sperm to fertilize eggs in the water column.”**

~ Also, this might be a good place to highlight that the plasticity utilized by B and NB may have been equally beneficial for B and NB before but that now egg quality might be suffering for B mothers due to continued impacts.

**Response: This has been revised to address the comment in the discussion, please see LN 378-384: “This apparent tradeoff in reproductive effort suggests plasticity in response to environmental changes and emphasizes the need for long-term studies to detect changes in sexual reproduction** [**(Levitan et al.**](https://paperpile.com/c/pW0cc0/cyNj0+oxLq)**, 2014; Hagedorn** [**et al.**](https://paperpile.com/c/pW0cc0/cyNj0+oxLq)**, 2016; Price et al., 2019; Schlesinger et al., 2019). While further examination of egg traits, such as total lipid content and composition of lipid classes, was beyond the scope of this study due to limited material available, larger egg volume could be beneficial in storing lipids and carbohydrates as well as increased surface area for slower sperm to fertilize eggs in the water column.”**

4.2 Parental effects on fertilization and offspring survivorship:

“In this study, bleached colonies had similar fertilization success to nonbleached colonies, the thermally tolerant individuals will likely have a stronger selective advantage as warming continues to weed out thermally sensitive adults and their offspring (Drury et al., 2022).”
~ I do not understand this statement, authors need to clarify more here. How can the B and NB colonies having similar fertilization success mean there is stronger selective advantage for NB adults and offspring? I understand why the authors are trying to link in natural selection towards more resilient individuals, but that is contrary to the results discussed in the beginning of this sentence. Especially since similar fertilization, number of eggs, and volume of eggs were found across both these groups for both years following the bleaching events. Rework this statement for clarity.

**Response: We have revised this section to:
LN 422-426 “While the coral colonies that experienced bleaching and recovered showed similar fertilization success rates compared to those that didn't bleach, it seems that the coral colonies with higher thermal tolerance have a stronger competitive edge. This advantage becomes more pronounced as rising temperatures and marine heatwaves continue to eliminate thermally sensitive adults and their offspring from the population.”**

“Beneficial cross-generational plasticity through maternal effects observed in offspring survivorship may be attributed to energetic provisioning through lipid reserves stored in the eggs and larvae (Jones et al., 2011; Padilla-Gamiño et al., 2013; Rivest et al., 2017), mitochondria (Dixon 15 et al., 2015), or vertical transmission of Symbiodinaceae from the parent into the eggs (Jones et al., 2010;Padilla-Gamiño et al., 2012; Quigley et al., 2016).”
~ This statement should be fleshed out more and clarified further rather than just being a list of semi-unrelated traits. For example, what is meant by “Mitochondria” in this list because it does not look like it is connected to energy reserves.

**Response: The sentence has been modified, see LN 432-437:**

**“Benefits of maternal effects could be associated with phenotypic traits that help overcome hurdles created by thermal stress such as energetic provisioning through lipid reserves stored in the eggs and larvae (Jones et al., 2011; Padilla-Gamiño et al., 2013; Rivest et al., 2017, Rogrigues and Padilla-Gamiño 2022), or vertical transmission of Symbiodinaceae from the parent to the eggs (Jones et al., 2010; Padilla-Gamiño et al., 2012; Quigley et al., 2016).”**

~ Also, Padilla-Gamiño et al., 2013 does not actually identify the symbionts in M. capitata so it would be more correct to cite Padilla-Gamiño et al. 2012 in PLoSOne instead, or better yet de Souza et al. 2022 since that has more recent and specific symbiont identifications for M. capitata in Kāne‘ohe Bay.
“In M. capitata, these symbionts are vertically transferred to the eggs creating offspring with different assemblages (Padilla-Gamiño et al., 2013) that could confer different physiological attributes to the offspring.”
~ This should also be Padilla-Gamiño et al., 2012 PLoSOne that looked at symbionts in eggs, rather than 2013.

**Response: We have revised the Padilla-Gamino references and have included Souza et al. 2022 to include the most recent studies on the Symbiodineaceae composition and distribution in Kāneʻohe Bay and the impacts of bleaching on symbiont composition.**

**Rocha de Souza Mariana, Caruso Carlo, Ruiz-Jones Lupita, Drury Crawford, Gates Ruth and Toonen Robert J. 2022 Community composition of coral-associated Symbiodiniaceae differs across fine-scale environmental gradients in Kāne‘ohe Bay. R. Soc. Open Sci.9212042212042**

“For example, Little et al. (2004) found that Acropora juveniles grew faster when infected with clade C than clade D, and Abrego et al. (2008)”
~ Same comment about symbiont names here. Look up the specific genera for these symbiont-host associations. It is not appropriate to call the symbionts by the antiquated “Clades” designations, also the authors should be consistent with how they reference the symbionts throughout the manuscript rather than changing between genera names and clade names.

**Response: We have modified this sentence, see LN 448-454:**

**“For example, Little et al. (2004) found that *Acropora* juveniles grew faster when infected with *Cladocopium* spp. than *Durusdinium* spp. (formerly clade C and D, respectively) clade C than clade D, and Abrego et al. (2008) showed enhanced physiological tolerance and higher ^14^C photosynthate incorporation in juveniles infected with *Cladocopium* spp. (clade C1).”**

“Our results suggest that nonbleached colonies have higher ecological fitness than their bleached counterparts and may benefit the maintenance of genetic diversity while guiding populations towards higher thermal tolerance in a warming ocean.”
~ Authors discuss the importance of the photosynthetic endosymbiont to the host and that this symbiont is vertically transmitted to the larvae by the mother. After disturbance the dominant Symbiodiniaceae community members can shift rapidly back to a previous non-disturbed community state (sometimes within a few months to a year, see references below this comment).

**Response: Yes, indeed. Rocha de Souza et al. (2022) found that following a bleaching event, the relative proportion of the heat-tolerant symbiont *Durusdinium* in *M. capitata* increased in most parts of the bay. Despite this increase, the overall composition of algal symbionts remained largely unchanged, and distinct regions of the bay retained their pre-bleaching compositions.**

It is important to know what symbionts were at play in B and NB parents (and the sampled larvae) before making any statements about what impacts these may have had on the larvae survivorship in the current study.
Were the symbionts ever identified for the parent colonies or subset of larvae to determine if any claims in this paragraph were relevant to the corals and findings of this study? If not, then the concluding sentence of this paragraph should be removed since no data collected by the authors corroborates the dominant symbiont of the mothers or larvae.
• Elder H, Million WC, Bartels E, Krediet CJ, Muller EM, Kenkel CD (2022) Long-term maintenance of a heterologous symbiont association in Acropora palmata on natural reefs. The ISME Journal
• Thornhill DJ, LaJeunesse TC, Kemp DW, Fitt WK, Schmidt GW (2006) Multi-year, seasonal genotypic surveys of coral-algal symbioses reveal prevalent stability or post-bleaching reversion. Marine Biology 148:711-722

**Response: We did not examine symbiont diversity in our samples. We have removed the concluding sentence of that paragraph.**

“M. capitata houses the endosymbionts Cladocopium spp. and Durusdinium spp., formerly Clade C and D, respectively.”
~ It is outdated and improper to list genera levels of specific symbiont-host associations. Since only one coral species is assessed in the current study the specific symbiont species that associate with this coral in this area should be listed instead (i.e., Durusdinium trenchii instead of “Durusdinium spp.”)

**Response: In Kāneʻohe Bay, Hawaiʻi, the specific symbiont species of *Cladocopium* spp. in *Montipora capitata* remains unknown. Recent unpublished work from the Coral Resilience Lab at HIMB have utilized ITS2 and psbA markers to confirm that in Hawaiʻi, *Montipora capitata* hosts *Durisdinium glynnii,* not *D. trenchii* as previously mentioned by the reviewer.**

4.3 Interventions for thermal tolerance:

“manipulation of coral-algal endosymbiosis”
~ the authors have not referenced the Symbiodiniaceae as “algae” yet in this manuscript or defined the use of “algae” to mean Symbiodiniaceae. For consistency in text and lack of confusion with readers this should be changed to reflect the terms used to reference the Symbiodiniaceae symbionts throughout the manuscript.

**Response: We have replaced algae or algal with Symbiodiniaceae symbionts throughout the manuscript.**

“Human interventions applying selective breeding in coral sexual propagation has been proposed as one of the viable options to maintain genetic diversity and increase resilience in restoration efforts (Barott et al., 2021; Epstein et al., 2003; van Oppen et al., 2015, 2017; NASEM, 2019; Hancock et al., 2021); however, feasibility to potentially scale up efforts remain limited and costly without full understanding of tradeoffs (Edwards et al., 2015; Chamberland et al., 2017).”
~ Need commas here like "Human interventions, such as selective breeding in coral sexual propagation, has been..."

**Response: We have edited this sentence to add clarity to the text. See LN 470-474: “Human interventions, such as selective breeding in coral sexual propagation, has been proposed as one of the viable options to maintain genetic diversity and increase resilience in restoration efforts** [**(Barott et al., 2021; Epstein et al., 2003; van Oppen et al.,**](https://paperpile.com/c/pW0cc0/zrY3+Bt8W+7xUK) **2015, 2017; NASEM, 2019; Hancock et al., 2021); however, feasibility to potentially scale up efforts remain limited and costly without full understanding of tradeoffs** [**(Edwards et al., 2015; Chamberland et al., 2017)**](https://paperpile.com/c/pW0cc0/Xz6D+8pFy)**.”**

~ The way this is written it sounds here like only thermally resistant colonies (NB colonies in this paper) should be considered for selective breeding, but thermal resilience is not the only trait corals need to survive anthropogenic climate change, which also causes increased disease prevalence and ocean acidification along with other factors. Since B adults have also been successful thus far, it is likely they have a different trait necessary for acclimating well to their home reefs, like disease resistance, greater trophic plasticity, or that they utilize different trade-offs to deal with thermal stress. The data here does not necessarily support selective breeding for thermal tolerance with individuals that do not bleach, but instead the preference for using eggs from NB parents when creating crosses following bleaching events. Therefore, the authors should consider making less definitive statements here.

**Response: This is a very good point. We have revised the sentence, please see LN 480-482: “These results are important to maximize restoration efforts through selective breeding by identifying candidate colonies in the natural environment or through manipulated stress tests and performing crosses using the gametes of resilient colonies.”**

Figures:

Figure 3: Reproductive output was higher in bleached colonies and had fewer abnormalities than NB colonies, how do the authors explain this in relation to their statements that the bleached colonies are less fit in the discussion?

Egg abnormality was not different between bleached and non-bleached colonies. Egg abnormality only significantly differed between years. In 2016, egg abnormality was higher than in 2017 regardless of parental bleaching history.

**Response: Higher reproductive output in bleached colonies may be the result of larger allocation of carbon to the eggs as it has been previously shown in M. capitata (Rodrigues and Padilla-Gamino 2022). Although bleached corals have more reproductive output (egg amount), it has been shown that sperm quality of bleached colonies is severely compromised by bleaching and thus, higher release of eggs does not necessarily translate into higher fitness (Henley et al. 2021).**

**See: Henley, E.M., Quinn, M., Bouwmeester, J. *et al.* Reproductive plasticity of Hawaiian Montipora corals following thermal stress. *Scientific Reports***

**11, 12525 (2021).** [**https://doi.org/10.1038/s41598-021-91030-8**](https://doi.org/10.1038/s41598-021-91030-8)

Figure 4: Panel B should include x-axis labels for clarity. It is confusing to look at a separate panel for the axis labels, especially since only those two panels in the figure share labels.

**Response: Thank you, we have added labels to the x-axis for Figure 4B to provide more clarity.**

Reviewer #2: PLOS ONE Review

General comments

Lenz and colleagues present results from experiments spanning multiple years to investigate the potential for multigenerational effects of thermal stress on the coral Montipora capitata. They found that while thermal stress impaired aspects of reproduction regardless of if a colony bleached or not, parental bleaching history was an important driver of offspring success. These findings supporting the concept that bleaching-resistant colonies could be targeted for selective breeding to produce offspring that to support the restoration of more thermally-tolerant coral populations. The authors do an excellent job of highlighting the importance of their findings and avoid overextending their conclusions beyond the scope of their study. Their findings are particularly timely given the increasing severity, prevalence, and mortality caused by thermal stress on coral reefs across the globe. I have relatively minor comments that are articulated below that I recommend addressing before the ms is published.

There are no line numbers on the submission, thus I have not included them below but did my best to describe where in the ms I am referring to by referencing paragraphs within sections.

**Response: We have included line numbers in the manuscript.**

Introduction: The introduction is well written and provides a thorough yet concise overview of how thermal stress can impact coral reproduction and population dynamics, while also highlighting the knowledge gap this paper addresses (intra- and intergenerational impacts of bleaching).

Paragraph (henceforth, P) 4: The sentence regarding high recruitment in Mo’orea after the 2016 thermal stress is incomplete/lacking details compared to the other examples. The examples presented in P3 and P4 provide clear links between thermal stress/bleaching and reproduction in corals. However, the Mo’orea example suggests that the minor 2016 bleaching event may have had the potential to affect reproductive output, but provides no concrete information that some corals were reproductively compromised. How it is currently written, one could conclude that the minor bleaching in 2016 did not impact reproduction. Suggest revising to better leverage this example for the introduction.

**Response:**

**For clarity, we have revised this paragraph and removed the sentence highlighting the high recruitment in Mo’orea.**

P5: Sentence with “~70% of reef corals on shallow reefs” – is there a reason that these corals are distinguished as reef corals? If not, remove to avoid confusion, or if there is an important distinction between reef corals and other corals, provide details for clarity.

**Response:**

**We have edited the sentence to add clarity.**

Methods

P2: Do the authors have any bleaching history of the colonies used in this study during 2014? If so, that would be worthwhile to include here or as supplemental information.

**Response:**

**While we agree that knowing the 2014 bleaching history of these colonies tagged during the 2015 bleaching event would be beneficial, we do not have this information. Based on work by Raphael Ritson Williams, Chris Wall, and Kristin Brown, we do know that there is a strong pattern of repeated bleaching in *M. capitata*.**

P5: For quantification of larval survivorship – was this recorded as the number of larvae remaining in an individual well-plate, or did this take into account the number of settlers (which is needed to quantify larval survivorship)? For example, if a well-plate started with 10 larvae, and 5 had settled after 7 days and 5 remained swimming, was this recorded as 100% survivorship (5 out of the 5 larvae remaining) or 50% survivorship (5 of the initial 10 placed in the well-plate)? This should be clarified in the methods section.

**Response:**

**For Figure 4D, we have revised to clarify this is the proportion of motile larvae. We have ensured this is consistent throughout the manuscript.**

P6: (second sentence) – revise for clarity. Mentions using a generalized mixed effects model twice in the same sentence, but is only referring to one model.
**Response: We have revised the sentence to be cleaner and only mention the glmer once.**

P7: The survivorship curve analysis includes days 23 and 27, but these days are not mentioned in the previous section regarding when the number of offspring alive were counted (days 6, 7, 28, 53, and 59).

**Response: Thank you for catching this error. For the survivorship curve, we have included day 7, 28, 53, and 59.**

P7: Was the effect of fragmentation included in any of the models to test if fragmenting impacted reproduction? Given the reported size of the fragments, there is not reason to believe there would be any impact, but this would be worth mentioning somewhere in the ms.

**Fragmentation was not included in the model because colonies were removed with a hammer and chisel, but previous work has shown that *M. capitata* (Cox and Padilla-Gamiño) can spawn despite fragmentation. Our fragments were large and had three days to acclimate in tanks before spawning. We provided clarification in the methods.**

Results

Discussion: The discussion is well written and thorough. The authors did a nice job of placing their findings in the context of previous work (e.g. reduction in no. of eggs/bundle), proposing potential hypotheses for the patterns they observed (and supporting evidence), expanding the discussion to broader concepts of population ecology, and explaining how these results can be used to inform interventions like selective breeding.

Figures

Figure 2: Suggest choosing a color palette that facilitates distinguishing between B and NB colonies. I can understand why these were chosen to look like healthy (NB) vs. bleached (B) colonies, but the colors are difficult to quickly distinguish.

**Response: We respectfully decline as these colors are representative of the changes observed in the field and want to maintain the color choices to represent the crosses.**

Figure 3: 3A -Lines are not appropriate for this type of plot – these data would be more clearly presented as a bar plot since each night of spawning is a discrete event, not continuous as the lines connecting the dots imply.

**Response: We have revised 3A to be barplots to distinguish the proportion of colonies that spawned and those that did not over the two spawning seasons in 2016 and 2017.**

3B-E: suggest adding a legend for the B and NB colors even though it is on the x-axis labels (and hidden in the 2016 panel of Figure 3A.
**Response: We have made the legend more clear in Figure 3A and believe this revision makes it more clear for 3B-E and would like to keep as is to avoid too much redundancy in the same image.**

Figure 4B: Add x-axis labels to this plot, it is difficult to follow them up from Fig 4C.
**Response: We have added the x-axis labels to 4B and 4D as well for clarity.**

4C: Error bars on stacked bar plots make for very busy figures that are difficult to interpret and take away from the data being shown. Suggest individual bars for each of the developmental stages for each cross – this would make the difference in developmental stages easily observable by looking at Fig 4C.

**Response: We have removed the error bar caps from Figure 4C and 4B for consistency and clarity.**

4D-F: Similar to Fig 4, these colors are similar and thus hard to distinguish, especially given the that these are small panels within a 6-panel figure. Suggest selecting a different color scheme.

**Response: We have kept the color scheme to be representative of the parental bleaching history.**

Reviewer #3: I enjoyed reading "Parental effects provide an opportunity for coral resilience following major bleaching events". I think the manuscript is sound and comprehensive look at colony fecundity, well-written, and almost ready for publication. My only reservation is some aspects of the discussion don't quite jive with the findings which I will detail below. However, I think this is good work and after a tidy up of some claims in the discussion, warrants publication. Despite my concerns with the discussion, I think the summary in the last paragraph "In our study... to balance reduced survivorship and settlement" to be a perfect summary of the findings.

Major concerns
*To me, the main result is that parental bleaching history did not significantly affect colony fecundity, the size of eggs, or eggs per bundle. If true, this runs counter to a lot of the discussion which emphasizes how bleaching affects reproductive capacity. For example on page 12, authors claims that this study "demonstrates the impact of bleaching on reproductive capacity" but the stated evidence doesn't show sig differences between B/NB (egg volume, eggs/bundle, abnormalities). Moreover, this carries over to the fertilization, survival, and settlement result where there seem to be no differences between larvae from B and NB colonies.

**Response: We have edited the discussion to highlight the maternal effects as an important result in our study. The significance of this study is that while we did not observe effects at the gamete level, the impacts of parental bleaching history became more apparent in the next generation.**

On pg 15, authors claim that NB colonies have higher fitness, but I don't see how the results of this study support that for the reasons above plus seemingly minor effects on survival (I didn't see the magnitude of maternal effect on survival mentioned... seems small).

**Response: We have removed the text where we suggest that NB has higher fitness.**

*Consider making maternal effects a larger focus of the discussion if this was the main sig result (and few outright B/NB differences).

**Response: We have edited the discussion to highlight the maternal effects as the main significant result.**

*Discussion seems to be a bit fast and loose when describing insignificant effects. A focus on effect size could help support your claims. For example on pg 12, authors claim that 'maternal effects are demonstrated from fertilization through survival' but the maternal effect on fertilization is insignificant and the effect on survival seems to be quite small (Fig 4E inset).

Same issue with "colonies have higher fitness" claim on pg 15.

**Response: We have deleted the text associated with "colonies have higher fitness.”**

*The sig. difference in proportion colonies spawned 2016 vs 2017 seems to be largely due to the third month, which is a light spawn in 2016 and no spawn in 2017. There seems to be little difference in months 1 and 2 between years. This may affect how the effects of 2015 bleaching are interpreted

*Where is there a significant maternal effect of NB/B? Do analyses need to be redesigned to test for maternal NB/B versus paternal NB/B?

Minor concerns
*Not sure if this is due to journal format, but Tables were not included in the submission which made it difficult to evaluate some claims
**Response: Thank you for notifying us, the tables were in a separate file and we did not realize those would not be provided in the review process.**

*I doubt it will change the conclusions, but technically, proportions and counts shouldn't use gaussian error distribution in linear mixed model

*pg11 Results section 3.2 p value 0.342 is in a different font
**Response: The font has been revised to be Times New Roman.**

*Consider shrinking the discussion section on interventions

**Response: After consideration, we decided to leave this section and not shorten the discussion on interventions as Reviewer #2 appreciated this section of the manuscript and found it important to highlight how our results can be applied and inform continued research on interventions like selective breeding.**

* Could be due to PDF conversion, but figures were blurry

**Response: Figures with high resolution have been submitted.**

* pg 12 please add 'for example' to describe what "delayed beneficial maternal effects might be" (you give more details later). Maybe maternal effects need to be earlier in the disc. and a bigger part of the manuscript.

**Response: We have included the following text on page 14.**

**Benefits of maternal effects could be associated with phenotypic traits that help overcome hurdles created by thermal stress such as energetic provisioning through lipid reserves stored in the eggs and larvae (Jones et al., 2011; Padilla-Gamiño et al., 2013; Rivest et al., 2017, Rogrigues and Padilla-Gamiño 2022), or vertical transmission of Symbiodinaceae from the parent to the eggs (Jones et al., 2010; Padilla-Gamiño et al., 2012; Quigley et al., 2016).**

* What led to 2016 vs 2017 differences in abnormality?
**Response: We have included the following text:
“This could be attributed to the proximity to the consecutive thermal stress events and the need for long-term recovery of the coral colonies.”**

* What could have led to B*B and NB*NB having faster development?
The lack of compatibility observed in crosses between B × NB and B × NB could potentially result from lineage crossing. However, in our study, we were unable to analyze the genetic composition of the parent organisms and we couldn't determine if they belonged to distinct parental lineages. Future studies should take into account parental lineages to better understand gamete compatibility, inheritance patterns and traits that can lead to increased genetic diversity or novel offspring phenotypes.

*Settlement rates seem a bit low. Is settlement typically <50% for this species?

**Response: Yes, these settlement rates are typical for *Montipora capitata* in Hawaii. Henley et al. (2021) found that settlement success in *M. capitata* ranged between 7% ±1.45% and 23% ± 4.94% in 2018 and 2019, respectively. Lager et al. (2020) found around 20% settlement success for *M. capitata* in Kāneʻohe Bay.**

**Henley EM, Quinn M, Bouwmeester J, Daly J, Zuchowicz N, Lager C, Bailey DW, Hagedorn M. Reproductive plasticity of Hawaiian *Montipora* corals following thermal stress. Sci Rep. 2021 Jun 9;11(1):12525. doi: 10.1038/s41598-021-91030-8. PMID: 34108494; PMCID: PMC8190081.**

**Lager, C.V., Hagedorn, M., Rodgers, K.S. and Jokiel, P.L., 2020. The impact of short-term exposure to near shore stressors on the early life stages of the reef building coral *Montipora capitata*. *PeerJ*, *8*, p.e9415.**

6. PLOS authors have the option to publish the peer review history of their article ([what does this mean?](https://urldefense.com/v3/__https:/journals.plos.org/plosone/s/editorial-and-peer-review-process*loc-peer-review-history__;Iw!!PvDODwlR4mBZyAb0!XuwFPj6fQDP7P6rzVi83CuVZHDP9fD2L2rF2-lSF_Y6KpeH5aJ0aUResU0mtRYL4UzKs92eWIEwloSQy$)). If published, this will include your full peer review and any attached files.


If you choose “no”, your identity will remain anonymous but your review may still be made public.


**Do you want your identity to be public for this peer review?** For information about this choice, including consent withdrawal, please see our [Privacy Policy](https://urldefense.com/v3/__https:/www.plos.org/privacy-policy__;!!PvDODwlR4mBZyAb0!XuwFPj6fQDP7P6rzVi83CuVZHDP9fD2L2rF2-lSF_Y6KpeH5aJ0aUResU0mtRYL4UzKs92eWIJm8vRHJ$).

Reviewer #1: No

Reviewer #2: No

Reviewer #3: No

[NOTE: If reviewer comments were submitted as an attachment file, they will be attached to this email and accessible via the submission site. Please log into your account, locate the manuscript record, and check for the action link "View Attachments". If this link does not appear, there are no attachment files.]

While revising your submission, please upload your figure files to the Preflight Analysis and Conversion Engine (PACE) digital diagnostic tool, [https://pacev2.apexcovantage.com/](https://urldefense.com/v3/__https:/pacev2.apexcovantage.com/__;!!PvDODwlR4mBZyAb0!XuwFPj6fQDP7P6rzVi83CuVZHDP9fD2L2rF2-lSF_Y6KpeH5aJ0aUResU0mtRYL4UzKs92eWIP5J-zPB$). PACE helps ensure that figures meet PLOS requirements. To use PACE, you must first register as a user. Registration is free. Then, login and navigate to the UPLOAD tab, where you will find detailed instructions on how to use the tool. If you encounter any issues or have any questions when using PACE, please email PLOS at figures@plos.org. Please note that Supporting Information files do not need this step.
